# A new look at phosphorus intake: what do we eat here is what they eat there?

**DOI:** 10.1590/2175-8239-JBN-2018-0231

**Published:** 2019-01-24

**Authors:** Christiane Ishikawa Ramos, Lilian Cuppari

**Affiliations:** 1 Universidade Federal de São Paulo, Escola Paulista de Medicina, São Paulo, SP, Brasil.; 2 Fundação Oswaldo Ramos, Hospital do Rim, São Paulo, SP, Brasil.

Managing hyperphosphatemia is one of the most significant challenges in the treatment of individuals with chronic kidney disease (CKD) particularly those on dialysis. Decreased excretion of phosphorus in individuals with CKD results in phosphorus overload, a condition that stimulates the synthesis of fibroblast growth factor 23 (FGF-23) and parathyroid hormone (PTH), two factors involved in the development of chronic kidney disease-mineral and bone disorder (CKD-MBD). CKD-MBD increases the risk for cardiovascular disease, death, and deteriorates quality of life.^1^

Organic and inorganic phosphorus can be found in a wide variety of foods. Animal-based foods, such as meat and dairy products, are among the main sources of organic phosphorus in omnivorous diets. The phosphorus present in these foods is more easily digested than the phosphorus from plant-based foods (> 70% vs. < 40%, respectively), in which most phosphorus is bound to phytate, a carbohydrate that cannot be digested by the enzymes in the human gastrointestinal tract, thus hampering the absorption of phosphorus.^2^ However, in modern societies excessive phosphorus intake has been attributed to processed foods containing "phosphorus additives" such as sausages, processed cheese, cola-based carbonated drinks, etc.^2^ Research indicates that these foods account for 30% of the energy intake of Brazilians.^3^ A study carried out in Rio de Janeiro found that processed food intake accounted for more than 37% of energy intake of elderly individuals on dialysis, a proportion higher than the one found for age-matched individuals without CKD.^4^ This is an alarming finding, since these foods offer easily absorbable inorganic phosphorus and may, therefore, contribute to the development of phosphorus overload in individuals with CKD. A study on nutrition education carried out in São Paulo in which processed foods were replaced for similar natural foods reported significant decrease in serum phosphate in patients on hemodialysis with hyperphosphatemia.^5^ These results illustrate the negative impact of phosphorus present in food additives on serum phosphate levels and the benefits of nutrition education strategies in the management of hyperphosphatemia.

Despite the data presented above, the intake of sources of phosphorus by patients with CKD in Brazil requires further analysis. In this context, the study performed by Nerbass et al.^6^ published in this issue of the Brazilian Journal of Nephrology is innovative since it explores the main food sources of phosphorus present in the diets of individuals on dialysis, in addition to offering the comparison between the intake pattern of these foods in individuals from the States of Tocantins and Santa Catarina. The relevance of the analysis lies on the continental proportions of the country and the many geographic, socioeconomic, and cultural differences between its regions, which may be reflected in the eating habits of the population.

The authors used a food frequency questionnaire designed to assess phosphorus intake and noted that, in general terms, the most frequent reported items were milk and beans. The frequency score for food sources of organic phosphorus was higher than the score for inorganic phosphorus, but only the inorganic phosphorus scores were associated with hyperphosphatemia, an indication of the potential negative effects of phosphorus-containing additives on individuals with CKD. When the two States were compared, Santa Catarina had a higher prevalence of hyperphosphatemia than Tocantins, although the patients underwent the same clinical protocol to manage CKD-MBD. Therefore, although the study did not include a quantitative assessment of phosphorus intake, differences in the food items reported in each State may explain the difference in serum phosphate levels seen in serum phosphate levels. Pork, dairy products, and sausages were more commonly eaten in Santa Catarina, whereas in Tocantins the main food items consumed were foods were beef and beans. The results reported by the authors suggest that the intake of inorganic phosphorus-containing food additives present in sausages and probably in dairy products was higher in Santa Catarina than in Tocantins.

The strategy adopted by Nerbass et al. to assess food intake is in line with current research practice current research practice that attempts to comprehend the relationship between diet and clinical outcomes based on food groups and eating habits instead of isolated nutrients. This approach has advantages since it considers synergies between foods, nutrients, and bioactive compounds, in addition to facilitating the communication of dietary advices between healthcare workers and patients. Furthermore, the assessment of isolated nutrients may be more susceptible to measurement errors, due to the limited information on phosphorus contents in food composition tables and processed food labels, which lead to the underestimation of phosphorus intake.

The study indicates that nutrition education strategies for the management of to hyperphosphatemia should be based on the particular eating habits of each individual population. Therefore, studies based on qualitative assessment of food intake that support the production of scientific evidence to guide more effective dietary interventions in the management of hyperphosphatemia, are still necessary.
